# *CMIP* and *ATP2C2* Modulate Phonological Short-Term Memory in Language Impairment

**DOI:** 10.1016/j.ajhg.2009.07.004

**Published:** 2009-08-14

**Authors:** Dianne F. Newbury, Laura Winchester, Laura Addis, Silvia Paracchini, Lyn-Louise Buckingham, Ann Clark, Wendy Cohen, Hilary Cowie, Katharina Dworzynski, Andrea Everitt, Ian M. Goodyer, Elizabeth Hennessy, A. David Kindley, Laura L. Miller, Jamal Nasir, Anne O'Hare, Duncan Shaw, Zoe Simkin, Emily Simonoff, Vicky Slonims, Jocelynne Watson, Jiannis Ragoussis, Simon E. Fisher, Jonathon R. Seckl, Peter J. Helms, Patrick F. Bolton, Andrew Pickles, Gina Conti-Ramsden, Gillian Baird, Dorothy V.M. Bishop, Anthony P. Monaco

**Affiliations:** 1 Wellcome Trust Centre for Human Genetics, University of Oxford, Oxford OX3 7BN, UK; 2 Speech and Hearing Sciences, Queen Margaret University, Edinburgh EH21 6UU, UK; 3 Department of Educational and Professional Studies, University of Strathclyde, Glasgow G13 1PP UK; 4 Department of Speech and Language Therapy, Royal Hospital for Sick Children, Edinburgh EH9 1LF, UK; 5 Department of Child and Adolescent Psychiatry, Institute of Psychiatry, London SE5 8AF, UK; 6 Department of Child Health, University of Aberdeen, Aberdeen AB25 2GZ, UK; 7 Developmental Psychiatry Section, University of Cambridge, Cambridge CB2 8AH, UK; 8 The Raeden Centre and Grampian University Hospitals Trust, Aberdeen AB2 4PE, UK; 9 Avon Longitudinal Study Parents and Children, Department of Social Medicine, University of Bristol, Bristol BS8 2BN, UK; 10 Division of Clinical Developmental Sciences St. George's University of London, London SW17 0RE, UK; 11 Department of Reproductive and Developmental Sciences, University of Edinburgh, Edinburgh EH9 1UW, UK; 12 Human Communication and Deafness, School of Psychological Sciences, University of Manchester, Manchester M13 9PL, UK; 13 Newcomen Centre, Guy's Hospital, London SE1 9RT, UK; 14 The Queen's Medical Research Institute, University of Edinburgh, Edinburgh EH16 4TJ, UK; 15 Department of Child and Adolescent Psychiatry and Medical Research Council Centre for Social, Developmental, and Genetic Psychiatry, Institute of Psychiatry, London SE5 8AF, UK; 16 Biostatistics Group, School of Epidemiology and Health Science, University of Manchester, UK; 17 Department of Experimental Psychology, University of Oxford, Oxford OX1 3UD, UK; 18 The SLI Consortium, UK

## Abstract

Specific language impairment (SLI) is a common developmental disorder characterized by difficulties in language acquisition despite otherwise normal development and in the absence of any obvious explanatory factors. We performed a high-density screen of SLI1, a region of chromosome 16q that shows highly significant and consistent linkage to nonword repetition, a measure of phonological short-term memory that is commonly impaired in SLI. Using two independent language-impaired samples, one family-based (211 families) and another selected from a population cohort on the basis of extreme language measures (490 cases), we detected association to two genes in the SLI1 region: that encoding c-maf-inducing protein (*CMIP*, minP = 5.5 × 10^−7^ at rs6564903) and that encoding calcium-transporting ATPase, type2C, member2 (*ATP2C2*, minP = 2.0 × 10^−5^ at rs11860694). Regression modeling indicated that each of these loci exerts an independent effect upon nonword repetition ability. Despite the consistent findings in language-impaired samples, investigation in a large unselected cohort (n = 3612) did not detect association. We therefore propose that variants in *CMIP* and *ATP2C2* act to modulate phonological short-term memory primarily in the context of language impairment. As such, this investigation supports the hypothesis that some causes of language impairment are distinct from factors that influence normal language variation. This work therefore implicates *CMIP* and *ATP2C2* in the etiology of SLI and provides molecular evidence for the importance of phonological short-term memory in language acquisition.

## Main Text

Developmental speech and language disorders are a heterogeneous group of childhood conditions with variable presentation and etiology. Together, they account for 40% of pediatric referrals^1^ and statements of educational need.^2^ The term *specific language impairment* (SLI) defines a category of speech and language disorders in which a profound language impairment represents the primary deficit.^2^ This disorder affects 5%–8% of preschool children^2^ and is highly heritable.^3^ Nonetheless, in contrast to other related developmental disabilities (e.g., dyslexia [MIM #127700] and attention deficit hyperactivity disorder [ADHD, MIM #143465]), relatively few genetic studies have been performed for SLI. SLI is a prototypical multifactorial disorder that is predicted to involve numerous genetic loci and environmental factors.^3^ Three primary sites of linkage have been described^4^, ^5^, the most robust of which is on chromosome 16q (SLI1, MIM #606711). This region is of interest because the linkage is highly specific to a single psychometric measure (nonword repetition).^4^, ^6^, ^7^ The test for nonword repetition involves the repetition of nonsensical words of increasing length and complexity and is regarded as a measure of phonological (speech sound) processing and short-term memory.^8^ Individuals with SLI typically perform particularly poorly on nonword repetition, even when their language difficulties have apparently resolved, leading to the postulation that a short-term memory deficit causes susceptibility to SLI^9^ by impairing the retention of novel verbal information.^10^ This paper incorporates two contingent investigations: an association screen of the SLI1 region in a cohort of language-impaired families and a subsequent replication study of detected association effects in an independent sample selected from the Avon Longitudinal Study of Parents and Children (ALSPAC) general-population cohort.^11^, ^12^

The association screen utilized 806 individuals from 211 families ascertained by the SLI Consortium (SLIC). This nuclear-family cohort was collected from five sites across the UK (The Newcomen Centre at Guy's Hospital, London; the Cambridge Language and Speech Project (CLASP)^13^; the Child Life and Health Department at the University of Edinburgh^14^; the Department of Child Health at the University of Aberdeen; and the Manchester Language Study^15^, ^16^) and included the families in whom the SLI1 linkage was originally identified. Ethical permission for each collection was granted by local ethics committees. SLIC families were all selected on the basis of a single proband with receptive and/or expressive language skills more than 1.5 SD below the normative mean for his or her age. A more detailed description of these samples and the exclusionary criteria applied to the SLIC collection can be found in previous publications.^4^, ^6^, ^7^

Genotyping for the association screen was performed in two phases with a combination of Sequenom and Illumina technologies. We performed an initial high-density screen involving 1906 SNPs to tag all 58 genes (including introns, exons, and 5 Kb 5′ and 2 Kb 3′ of coding sequences) mapped to the 10.29 Mb SLI1 region of linkage (D16S3138–D16S413. Chromosome 16 position 76.16 Mb–86.45 Mb [B35]). Haplotype blocks were built within Haploview^17^ via the Gabriel method.^18^ Any between-block gap that was more than 15 Kb in size was tagged with the Tagger algorithm. Two genes that mapped to the region (*CDH13* [MIM #601364] and *WWOX* [MIM #605131]) were found to be larger than 1 Mb in size. For these two genes, blocks were built to cover the exonic regions only. Any region containing a SNP that met our predefined significance threshold (p < 0.001 in any one analysis or p < 0.01 across both analyses) was then supplemented with additional markers in a follow-up panel that included 138 SNPs, eight of which had previously been genotyped. Both phases of genotyping were completed prior to the replication study and were subjected to consistent quality-control procedures. The total genotype mismatch rate was 0.73% for duplicated SNPs and 0.76% for duplicated samples. Across both phases, 261 (12.7%) of SNPs were excluded at the quality-control stage. These included SNPs with a genotype rate of <80%, a minor-allele frequency of <2.5%, SNPs with unusual Beadstudio cluster patterns (Illumina) or atypical peaks in MassArray TyperAnalyser (Sequenom), SNPs with a GenTrain score of <0.5 (Illumina), and markers that showed consistent bad inheritances (>10 errors after data clean up). Across the entire region, the merged data set consisted of, on average, one SNP every 6.4 Kb. Across the known genes, there was on average one SNP every 4.5 Kb, and the largest remaining gap between blocks was 19,579 bp. Details of SNP coverage can be found in Table S1. Q-Q plots can be found in Figure S1. Given the consistent linkage between SLI1 and nonword repetition, all association analyses were based upon this measure. Our principal analysis involved the variance-components modeling of 28-item nonword repetition scores^8^ within 211 SLIC families (ao option) as a quantitative trait and was performed within QTDT.^19^ In addition, we performed a categorical case-control allelic test of association within PLINK.^20^ In this case-control analysis, SLIC individuals with low nonword-repetition scores (>2 SD below population mean, n = 79) were chosen as cases, and family members with above-average performance (>0.5 SD above population mean, n = 71) were used as controls. To avoid interdependence, we selected only one case or control from each family unit.

**Figure 1:**
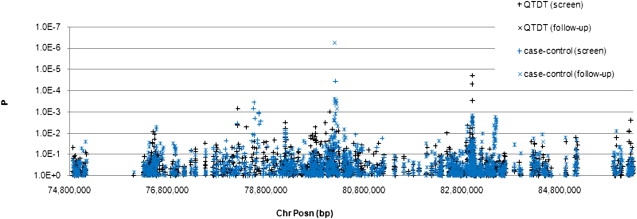
Association in SLIC Cohort Association results for family-based quantitaive analysis and case-control analysis of nonword repetition across the SLI1 region. In the case-control analysis, cases and controls were selected on the basis of their nonword-repetition performance (see text). Gaps in data represent regions where there are no mapped genes. SNPS included in the screen genotype panel are shown as +, and SNPs included in the follow-up genotype panel are shown as x.

The initial screen involved 1678 SNPs, of which thirteen (0.77%) exceeded our significance threshold, highlighting two primary regions of association (Table 1 and Figure 1). The follow-up panel chiefly included SNPs in these two regions and supported the association seen in the screen while reducing the evidence for association at other loci (Table 2 and Figure 1). Of the 105 SNPs tested in the follow-up panel, five (4.8%) were found to be significantly associated (Table 2 and Figure 1). The first identified cluster of association lay across 26 Kb (exons 2–4) of the *CMIP* gene (MIM #610112; seven significant SNPs, minP = 5 × 10^−7^). This gene encodes an adaptor protein and has two isoforms, the shorter of which is involved in cell signaling pathways and is upregulated in minimal change nephrotic syndrome (MCNS), a childhood kidney disease.^21^ Little is known about the function of the longer transcript. Both isoforms are expressed in the brain.^21^ The second region of association was observed between exons 7 and 12 (10.8 Kb) of the *ATP2C2* gene (six significant SNPs, minP = 2 × 10^−5^). This gene is one of two secretory-pathway Ca^2+^-ATPases (SPCAs) that move cytosolic calcium and manganese ions into the golgi.^22^ Its expression is limited to the brain, testis, gastrointestinal tract, and respiratory tissues and mammary, salivary, and thyroid glands.^22^ In the mammary gland, *ATP2C2* expression facilitates the secretion of Ca^2+^ into casein micelles during lactation.^23^

**Table 1:** Significant Association in the SLIC Association Screen

SNP	Chromosome Position (bp – B36)	Gene	Alleles (A1/A2)	A1 CEPH Frequency	Typed Strand	p Quant	Effect Size	Heritability	p Emp QTDT	p Case-Cont	Frequency of A1 Cases	Frequency of A1 Controls	Odds ratio (95% CI)	p Emp PLINK
rs8051754	78,554,834	intergenic	T/C^∗^	0.46	−	0.0931	−0.28 ± 0.11	0.019	0.0892	0.0007^∗^	0.64	0.85	3.1 (1.6–6.0)	0.0018^∗^
rs4417561	78,568,860	intergenic	G^∗^/C	0.26	−	0.0244	−0.30 ± 0.11	0.022	0.0252	0.0004^∗^	0.37	0.15	3.2 (1.7–6.3)	0.0011^∗^
rs2316184	79,204,885	*CDYL2*	G/A^∗^	0.14	+	0.0032^∗^	-0.48 ± 0.12^∗^	0.045	0.0034^∗^	0.0096^∗^	0.15	0.30	2.5 (1.2–4.9)	0.0126
rs12927866	80,209,823	*CMIP*	A/G^∗^	0.47	−	0.4104	−0.27 ± 0.10	0.019	0.3581	0.0003^∗^	0.29	0.49	2.4 (1.5–3.9)	0.0004^∗^
rs4265801	80,222,553	*CMIP*	T^∗^/G	0.43	+	0.3446	−0.09 ± 0.09	0.030	0.5065	4 × 10^−5^^∗^	0.61	0.29	3.9 (2.0–7.6)	0.0393^∗^
rs7201632	80,234,949	*CMIP*	C/T^∗^	0.49	+	0.8966	−0.25 ± 0.09	0.017	0.7975	0.0004^∗^	0.36	0.56	2.3 (1.4–3.7)	0.0004^∗^
rs3785054	82,918,978	*WFDC1*	C^∗^/T	0.36	−	0.0044^∗^	−0.29 ± 0.10^∗^	0.019	0.0033^∗^	0.0089^∗^	0.34	0.20	2.0 (1.2–3.4)	0.0102
rs8053211	83,011,254	*ATP2C2*	A^∗^/G	0.46	+	5 × 10^−5^^∗^	−0.38 ± 0.09^∗^	0.040	3 × 10^−5^^∗^	0.0014^∗^	0.61	0.43	2.1 (1.3–3.3)	0.0029^∗^
rs11860694	83,014,948	*ATP2C2*	C^∗^/G	0.54	−	2 × 10^−5^^∗^	−0.37 ± 0.09^∗^	0.039	9 × 10^−6^^∗^	0.0018^∗^	0.61	0.43	2.1 (1.3–3.3)	0.0027^∗^
rs16973771	83,018,079	*ATP2C2*	G/A^∗^	0.48	−	0.0003^∗^	−0.35 ± 0.09^∗^	0.034	0.0006^∗^	0.0025^∗^	0.34	0.51	2.0 (1.3–3.2)	0.0036^∗^
rs2875891	83,021,410	*ATP2C2*	T/C^∗^	0.44	+	0.0057^∗^	−0.34^∗^ ± 0.10^∗^	0.031	0.0063^∗^	0.0022^∗^	0.30	0.47	2.1 (1.3–3.4)	0.0026^∗^
rs8045507	83,022,078	*ATP2C2*	T/C^∗^	0.48	−	0.0017^∗^	−0.33 ± 0.09^∗^	0.029	0.0020^∗^	0.0022^∗^	0.34	0.51	2.1 (1.3–3.3)	0.0028^∗^

Three significant SNPs fell within the *CMIP* gene, and five fell within *ATP2C2*. The remaining four significant SNPs were either intergenic or isolated signals of association. SNP alleles are given with the minor allele in the SLIC sample first. Putative risk alleles are marked with an asterisk. P Quant gives the p value for the quantitative, family-based analysis. p case-cont gives the p value for the case-control analysis. p values <0.01 are marked with an asterisk. The odds ratios indicate the ratio of case/control odds for each additional copy of the putative risk allele. Odds ratios were calculated within PLINK. The effect size is the estimated effect of each risk allele on the nonword repetition score (in SD ± SE). Effect sizes were calculated with MERLIN. Heritability gives the proportion of total variance explained by the SNP. Heritability estimates were calculated with MERLIN. The p Emp column gives empirical p values for the given SNP; these values were derived from permutations within QTDT or PLINK.

**Table 2:** Significant Association in the SLIC Cohort with the Follow-up Panel

SNP	Chromosome Position (bp – B36)	Gene	Alleles (A1/A2)	A1 CEPH Frequency	Typed Strand	P Quant	Effect Size	Heritability	p Emp QTDT	p Case-Cont	Frequency of A1 Cases	Frequency of A1 Controls	Odds Ratio (95% CI)	p Emp PLINK
rs6564903	80,211,158	*CMIP*	C^∗^/T	0.48	+	0.1279	−0.37 ± 0.10	0.038	0.1225	5 × 10^−7^^∗^	0.79	0.38	3.5 (2.1–5.9)	1 × 10^−6^^∗^
rs3935802	80,219,068	*CMIP*	G^∗^/C	0.46	−	0.2667	−0.31 ± 0.10	0.025	0.2486	0.0003^∗^	0.71	0.49	2.5 (1.5–4.2)	0.0006^∗^
rs16955705	80,230,851	*CMIP*	C/A^∗^	0.50	+	0.3916	−0.25 ± 0.10	0.017	0.3627	0.0003^∗^	0.31	0.54	2.6 (1.5–4.4)	0.0003^∗^
rs4243209	80,247,592	*CMIP*	C/T^∗^	0.22	+	0.0065^∗^	−0.42 ± 0.12	0.027	0.0043^∗^	0.0007^∗^	0.11	0.26	3.0 (1.6–5.8)	0.0012^∗^
rs12149426	83,022,607	*ATP2C2*	A/C^∗^	0.26	+	0.0064^∗^	−0.31 ± 0.12	0.017	0.0082^∗^	0.0082^∗^	0.14	0.27	2.3 (1.2–4.2)	0.0039^∗^

Of the 105 SNPs analyzed in the follow-up panel, 16 lay in *CMIP*, 76 lay in the *ATP2C2* gene, and the remaining 13 lay in other regions that had shown association in the screen (see Table 1). Eight SNPs were genotyped in both the screen and follow-up panels. All of these markers showed some evidence of association in the screen phase (p < 0.01) but had genotype success rates of <95%, and none lay within *CMIP* or *ATP2C2*. Each of the duplicated SNPs showed increased success rates and decreased association levels in the follow-up panel. SNP alleles are given with the minor allele in the SLIC sample first. Putative risk alleles are marked with an asterisk. p Quant gives the p value for the quantitative, family-based analysis. p case-cont gives the p value for the case-control analysis. p values <0.01 are shown in bold. The odds ratios indicate the ratio of case/control odds for each additional copy of the putative risk allele. Odds ratios were calculated within PLINK. The effect size is the estimated effect of each risk allele on the nonword-repetition score (in SD ± SE). Effect sizes were calculated with MERLIN. Heritability gives the proportion of total variance explained by the SNP. Heritability estimates were calculated with MERLIN. The p Emp column gives empirical p values for the given SNP; these values were derived from permutations within QTDT or PLINK.

Three lines of evidence indicate that the associations at *CMIP* and *ATP2C2* represent separate effects. First, we did not see any indication of long-range linkage disequilibrium between the two loci (which lie almost 3 Mb apart) in the SLIC cohort or public data (Figure S2). Second, the inclusion of a *CMIP* covariate in the linkage or association model did not affect the level of linkage or association seen at *ATP2C2* (or vice versa for *ATP2C2* covariates) (Figure S3). Finally, in a stepwise regression model, the group mean for SLIC individuals carrying a double-risk genotype was found to be significantly lower than those who were homozygous for risk at a single locus (p = 3.7 × 10^−6^, Table 3). In this model, the group mean for double-risk individuals was 15.8 points (1.05 SD) below that of individuals carrying nonrisk variants at both loci (Table 3). We therefore propose that *CMIP* and *ATP2C2* independently regulate nonword repetition performance and together underlie the linkage seen between SLI and chromosome 16.

**Table 3:** Nonword-Repetition Group Means for *CMIP* and *ATP2C2* Risk Variants

	Genotype (Number of Risk Alleles)	Single SNP	rs6564903 (*CMIP*)		
			TT (0)	CT (1)	CC (2)
Single SNP			96.62	92.57	86.30
rs11860694 (*ATP2C2*)	GG (0)	96.54	99.14	99.85	89.65
	CG (1)	91.77	99.40	93.10	85.84
	CC (2)	87.03	88.44	88.33	83.32

The effects of *CMIP* (rs6564903) and *ATP2C2* (rs11860694) on nonword-repetition performance were modeled as additive effects within a regression framework in the R package. This regression model included all available SLIC children with genotype and nonword-repetition data (n = 503). Group means were calculated for each SNP in isolation (“Single SNP” entries) and in combinations of genotypes (3 × 3 grid) across risk SNPs. Note that individuals carrying combinations of risk alleles performed significantly worse than those carrying risk variants at a single locus. Nonword-repetition scores are age adjusted and standardized against normal population controls with a mean of 100 and a SD of 15.

**Figure 2:**
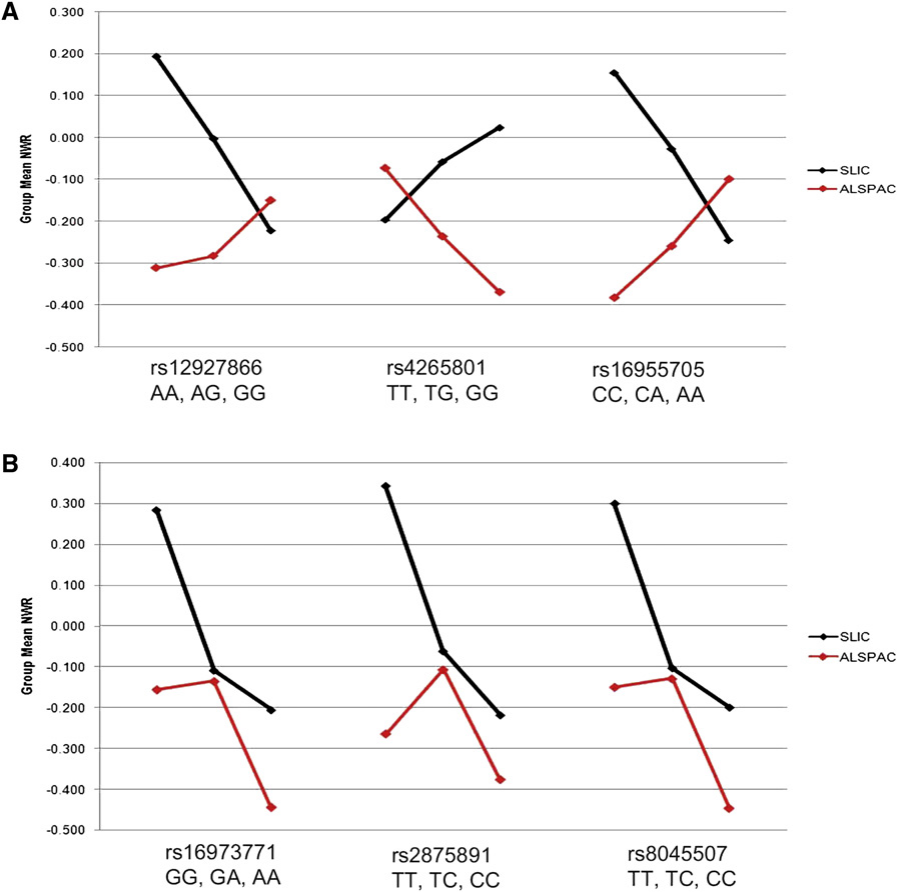
Nonword-Repetition Means for *CMIP* and *ATP2C2* in SLIC and Replication Cohorts (A) CMIP. (B) ATP2C2. All means are for age- and sex-adjusted nonword-repetition scores standardized with a mean of 0 and a SD of 1. The three *CMIP* SNPs (rs12927866, rs4265801, and rs16955705) show genotype trends in the opposite direction from SLIC (A), whereas the three *ATP2C2* SNPs (rs16973771, rs2875891, and rs8045507) show genotype trends in the same direction as SLIC (B).

Our replication sample consisted of 490 cases selected from the Avon Longitudinal Study of Parents and Children (ALSPAC) cohort.^11^, ^12^ This is a general-population sample that follows the development of 14,062 live-born individuals born in the southwest of England. The ALSPAC group periodically performs an assessment of the development of consenting individuals, and these measurements include tests of language ability. Informed written consent was obtained from the parents at the time of enrolment. Ethical approval for the study was obtained from the ALSPAC Law and Ethics Committee and the Local Research Ethics Committees. Because the current study focuses upon language impairment, we selected individuals from the lower extreme of language-related phenotype distributions (Children's Communication Checklist (CCC)^24^ and Wechsler Objective Language Dimensions (WOLD)^25^) for our replication sample. This included 665 individuals (10.3%) with a CCC pragmatic composite 1–3 SD below the ALSPAC population mean (123 ≤ × ≤ 145) or a WOLD listening comprehension score ≥2 SD below the ALSPAC population mean (≤3). Of these individuals, 490 had completed a 12-item nonword repetition test. Because the genotyping in the replication sample was restricted to a single individual from each family, we performed a quantitative association analysis within PLINK^20^ by using nonword repetition in a linear-regression framework. In addition, we used PLINK^20^ to carry out a case-control analysis analogous to that described for SLIC. We selected cases and controls from the extremes of the nonword repetition performance distribution of the 490 selected individuals. As expected, given the extreme nature of the language impairment in the SLIC samples, the distribution of nonword repetition differed between the SLIC and ALSPAC cohorts. Therefore, in the replication cohort, the cut-offs used for cases and controls were less extreme than those applied for the association screen. Cases were selected from the identified replication sample to have nonword repetition scores ≥1 SD below the general-population mean (n = 112), and controls had nonword repetition scores ≥1 SD above the general-population mean (n = 72). Data were analyzed for three *CMIP* and three *ATP2C2* SNPs (rs12927866, rs4265801, and rs16955705; and rs16973771, rs2875891, and rs8045507, respectively), and significant associations (p < 0.05) were seen for two *CMIP* and two *ATP2C2* SNPs (Table 4 and Figure 2). Regression trends for *ATP2C2* followed those seen in SLIC, replicating the previously described association. Association to *CMIP* was in an opposite direction from that described above (Table 4 and Figure 2). Although this result might represent a type I error, the consistency of significant association in light of the low number of SNPs tested supports a role for *CMIP*. Associations can occur in opposite directions if the relationship between the observed and causal variants differs between populations.^26^ This is particularly true if multiple risk loci interact in an additive or multiplicative fashion^26^, as is predicted for *CMIP*. Identification of the causal variant will enable the further characterization of the relationship between risk variants in different populations.

**Table 4:** Association in the Replication Cohort

SNP	Chromosome Position (bp – B36)	Gene	Alleles (A1/A2)	SLIC Risk Allele	A1 CEPH Frequency	Typed Strand	p Quant	Effect Size	p Case-Cont	Frequency of A1 Cases	Frequency of A1 controls	Odds Ratio (95% CI)
rs12927866	80,209,823	*CMIP*	T/C	C	0.47	+	0.1623	−0.08	0.0955	0.39	0.30	1.5 (0.9-2.3)
rs4265801	80,222,553	*CMIP*	T/G^∗^	T	0.43	+	0.0182^∗^	−0.15	0.0214^∗^	0.43	0.56	1.6 (1.1-2.5)
rs16955705	80,230,851	*CMIP*	C^∗^/A	A	0.50	+	0.0238^∗^	−0.14	0.0257^∗^	0.48	0.36	1.6 (1.1-2.5)
rs16973771	83,018,079	*ATP2C2*	C/T^∗^	T	0.48	+	0.0079^∗^	−0.14	0.0135^∗^	0.32	0.45	1.7 (1.1-2.7)
rs2875891	83,021,410	*ATP2C2*	T/C	C	0.44	+	0.0668	−0.06	0.0802	0.29	0.37	1.5 (1.0-2.3)
rs8045507	83,022,078	*ATP2C2*	A/G^∗^	G	0.48	+	0.0058^∗^	−0.15	0.0110^∗^	0.31	0.44	1.8 (1.1-2.7)

SNP alleles are given with the minor allele first. Putative risk alleles in the replication cohort are marked with an asterisk. p Quant shows the p value for the quantitative analysis. p < 0.05 are highlighted in bold. The odds ratio indicates the ratio of case/control odds for each additional copy of the putative risk allele. The 95% confidence intervals for the odds ratios of all significantly associated SNPs exceeded 1.0. The effect size is the estimated effect of each risk allele on the nonword-repetition score (in SD).

Given the partial replication of association, we investigated whether the primary associated SNPs in *ATP2C2* and *CMIP* had an effect upon additional language- and memory-related measures (Table S2). In SLIC, we found borderline association for *ATP2C2* with measures of receptive language (oral directions^27^ [p = 0.006], word classes^27^ [p = 0.04], and comprehension^28^ [p = 0.03]), expressive language (formulating sentences^27^ [p = 0.04]), and vocabulary^28^ (p = 0.04). In the replication cohort, aside from nonword repetition, we only observed borderline association between *ATP2C2* and counting span, a measure of working memory (p = 0.01). In the replication sample, nonword repetition performance had been scored according to the number of syllables the nonword contained. For both *CMIP* and *ATP2C2*, the majority of association came from the five-syllable nonwords (p = 0.016 and p = 6 × 10^−4^, respectively) (Table S2). In neither sample did we observe association to reading-related tasks, which have been reported to show linkage to SLI1.^6^ Nor did we find any association to digit span^28^ or recalling sentences,^27^ two measures that have a high memory load. This is consistent with the finding that nonword repetition correlates with SLI to a higher degree than other short-term memory tests (e.g., digit span). The sensitivity of nonword repetition to SLI could be because it places heavier demands on processing of speech sounds than other memory tests as a result of the child's having to perceive and produce an unfamiliar sequence.^29^ It is important to note that, although nonword repetition is a good marker for SLI, poor performance on nonword repetition is not a perfect correlate of this disorder.^30^ In our study, 50% of SLIC probands performed poorly (>1 SD below the expected population mean) on nonword repetition, but a significant number (27%) scored above the expected population mean. These findings support recent opinion that deficits across multiple domains are required to cause persistent language impairments.^31^

A recent genome-wide association study of ADHD listed a SNP (rs10514604; p = 8 × 10^−7^) in *ATP2C2* within the top 30 significant associations.^32^ Despite distinct defining characteristics, ADHD and SLI show a high level of comorbidity both with each other^32^ and with disorders such as developmental coordination disorder, speech-sound disorder (SSD; MIM #608445), and dyslexia.^33^, ^34^, ^35^ For example, individuals with SLI, SSD, ADHD, or dyslexia often present with linguistic deficits and impairments in short-term memory.^33^ It has therefore been suggested that certain aspects of these disorders might share a common etiology. Given the high levels of co-occurrence, we did not exclude children affected by ADHD and dyslexia from our study samples. However, in some of our SLIC samples, data were available for the presence of hyperactivity, coordination, and reading problems. From this, we estimate that approximately one-third of our SLIC samples showed some evidence of ADHD or developmental coordination disorder and that approximately one-half of our probands had reading problems. In the entire ASLPAC sample, 1.3% of individuals met criteria for ADHD. In the selected ALSPAC replication sample, the rate of ADHD increased to 3.7%. Thus, as expected, it is clear that the rate of developmental disorders across our cohorts is elevated over that expected in a population sample. Nonetheless, the association detected in our samples shows a strong correlation to nonword-repetition ability which has repeatedly been shown to be a strong indicator of language impairment.^9^, ^10^ Furthermore, in ADHD samples, performance on the nonword-repetition task is correlated with linguistic ability rather than the presence of hyperactivity.^33^, ^36^ Thus, we conclude that variants in *ATP2C2* might account for shared aspects of the linguistic deficit in SLI and ADHD. Given this possibility, we also postulate that *ATP2C2* might contribute to phonological short-term memory in other developmental disorders.

Finally, we investigated the effects of *ATP2C2* and *CMIP* on nonword-repetition performance at the population level. Across the entire unselected ALSPAC population (n = 3612), there was no evidence for quantitative association between nonword-repetition ability and either locus (minP = 0.48). Moreover, there were no differences in allele frequency for *ATP2C2* or *CMIP* SNPs between either SLIC or replication-sample individuals and unselected European population controls (data not shown). Taken together, these data indicate that *ATP2C2* and *CMIP* do not modulate nonword-repetition performance across the entire population, nor, in isolation, do they cause a predisposition to SLI. Instead, we propose that when combined with additional, as-yet-unidentified, susceptibility factors (either genetic or environmental), variants in *ATP2C2* and *CMIP* have a detrimental effect upon nonword repetition performance and thus heighten the risk of developmental language impairments. This situation demonstrates a fundamental principle often overlooked in the mapping of complex disorders: that genetic variants might have selective effects in specific populations depending upon the genetic and environmental background. The question as to whether SLI constitutes a qualitatively distinct disorder caused by abnormal development of language abilities or merely represents the tail end of normal linguistic development is a matter of recent debate.^37^ Although the absence of association in our population sample could reflect insufficient sample sizes or the insensitivity of psychometric tests to quantify variation beyond the lower extremes of the spectrum, it is obvious that the effects of *ATP2C2* and *CMIP* upon nonword-repetition performance are particularly pertinent to individuals with language difficulties. As such, this investigation provides molecular evidence that, at least in terms of the effects described here, SLI represents a distinct disorder caused by genetic variants discrete from those that influence language ability in the general population.

In summary, we have used a positional fine-mapping approach to demonstrate association between *ATP2C2* and *CMIP* and nonword repetition performance across two independent language-impaired populations. We propose that variants in both loci combine to modulate nonword-repetition performance in language-impaired populations. Both genes are expressed in the brain and represent good candidates for language- and memory-related processes. *ATP2C2* is involved in the translocation of cytosolic calcium and manganese ions to the golgi.^22^ Calcium homeostasis is important for the regulation of many neuronal processes, including working memory, synaptic plasticity, and neuronal motility^38^, and manganese dysregulation has been linked to Parkinsonism (MIM #168600), Alzheimer disease (MIM #104300), and disordered memory.^39^ The functional role of *CMIP* is less defined, but it is known to interact with filamin A (MIM #300017)^40^ and the NF-kappaB subunit RelA (MIM #164014).^41^ The filaminA protein is involved in the reorganization of the actin cytoskeleton, which is of importance in the formation of the dendritic spine.^40^ The NF-κB family of transcription factors plays a central role in many neuronal processes, including synaptic activity and memory formation, and members of this family have been implicated in neurodegenerative disorders.^42^ Further characterization of the observed associations has enabled us to infer that SLI represents a qualitatively distinct disorder caused by a combination of genetic variants that disrupt multiple pathways important to the development of language. It is anticipated that the functional characterization of *ATP2C2* and *CMIP* will promote a better understanding of the molecular basis of language acquisition and aid in the diagnosis and treatment of individuals affected by language disorders.

## Web Resources

The URLs for data presented herein are as follows:

Illumina, www.illumina.com/
Sequenom, http://www.sequenom.com/
GE Healthcare, http://www6.gelifesciences.com/
Online Mendelian Inheritance in Man (OMIM), http://www.ncbi.nlm.nih.gov/Omim
Tagger, http://www.broad.mit.edu/mpg/tagger/
Haploview, http://www.broad.mit.edu/mpg/haploview/
QTDT, http://www.sph.umich.edu/csg/abecasis/QTDT/
PLINK, http://pngu.mgh.harvard.edu/∼purcell/plink/
MERLIN, http://www.sph.umich.edu/csg/abecasis/Merlin/
PEDSTATS, http://www.sph.umich.edu/csg/abecasis/PedStats/
HAPMAP, http://www.hapmap.org/
R, http://www.r-project.org/
The Monaco Group at the Wellcome Trust Centre for Human Genetics (Neurogenetics), http://www.well.ox.ac.uk/monaco/
ALSPAC, http://www.bristol.ac.uk/alspac/
Manchester Language Study, http://www.manchesterlanguagestudy.co.uk/

## Supplemental Data

Document S1. Three Figures and Two Tables
