# Supplementary material for: CMIP and ATP2C2 Modulate Phonological Short-Term Memory in Language Impairment
Source: Am J Hum Genet. 2009 Aug 14;85(2):264–72. doi: 10.1016/j.ajhg.2009.07.004 (PMC2725236; doi:10.1016/j.ajhg.2009.07.004)
Supplement: Document S1. Three Figures and Two Tables [file mmc1.pdf]

The American Journal of Human Genetics Volume *85*

Supplemental Data

CMIP and *ATP2C2* Modulate Phonological Short-Term Memory in Language Impairment

Dianne F. Newbury, Laura Winchester, Laura Addis, Silvia Paracchini, Lyn-Louise Buckingham, Ann Clark, Wendy Cohen, Hilary Cowie, Katharina Dworzynski, Andrea Everitt, Ian M. Goodyer, Elizabeth Hennessy, A. David Kindley, Laura L. Miller, Jamal Nasir, Anne O'Hare, Duncan Shaw, Zoe Simkin, Emily Simonoff, Vicky Slonims, Jocelynne Watson, Jiannis Ragoussis, Simon E. Fisher, Jonathon R. Seckl, Peter J. Helms, Patrick F. Bolton, Andrew Pickles, Gina Conti-Ramsden, Gillian Baird, Dorothy V.M. Bishop, and Anthony P. Monaco

FIGURE S1 – Q-Q plots for SLIC genotyping

Dots represent observed chi2 against expected chi2 distribution, lines represent lines of best fit for the data shown. Dashed line represents line of best fit for QTDT, dotted line represents line of best fit for case-control and solid line represents the expected chi2 distribution. The correlation between expected and observed chi2 (R^2) was 0.9928 for the QTDT analyses and 0.9825 for the case-control analyses.

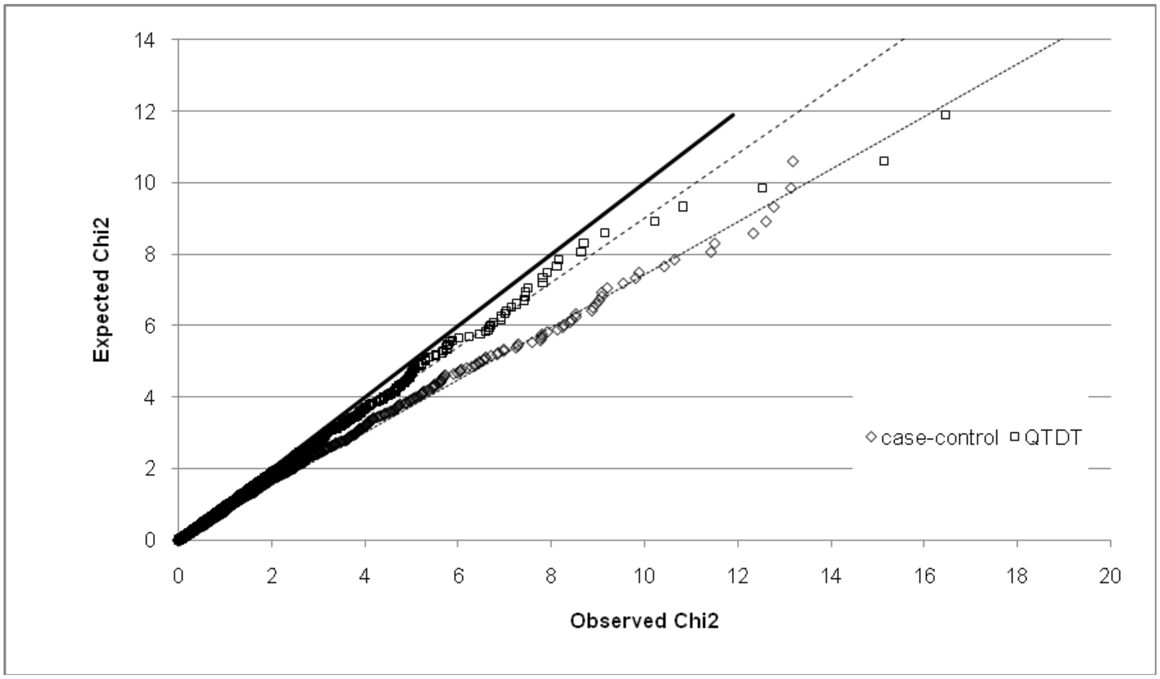

FIGURE S2 – Linkage disequilibrium between *CMIP* and *ATP2C2* in SLIC samples

LD between *CMIP* and *ATP2C2* in the SLIC samples. The arrows indicate the position of each gene, the triangles indicate the block of association found in each gene and the boxed region represents the region of LD between the 2 association blocks. LD is plotted as r^2 given by Haploview. Black squares show pairs of SNPs in perfect correlation (r^2=1), white squares represent pairs of SNPs with no correlation (r^2=0) and grey squares represent an intermediate level of correlation (0<r^2<1).

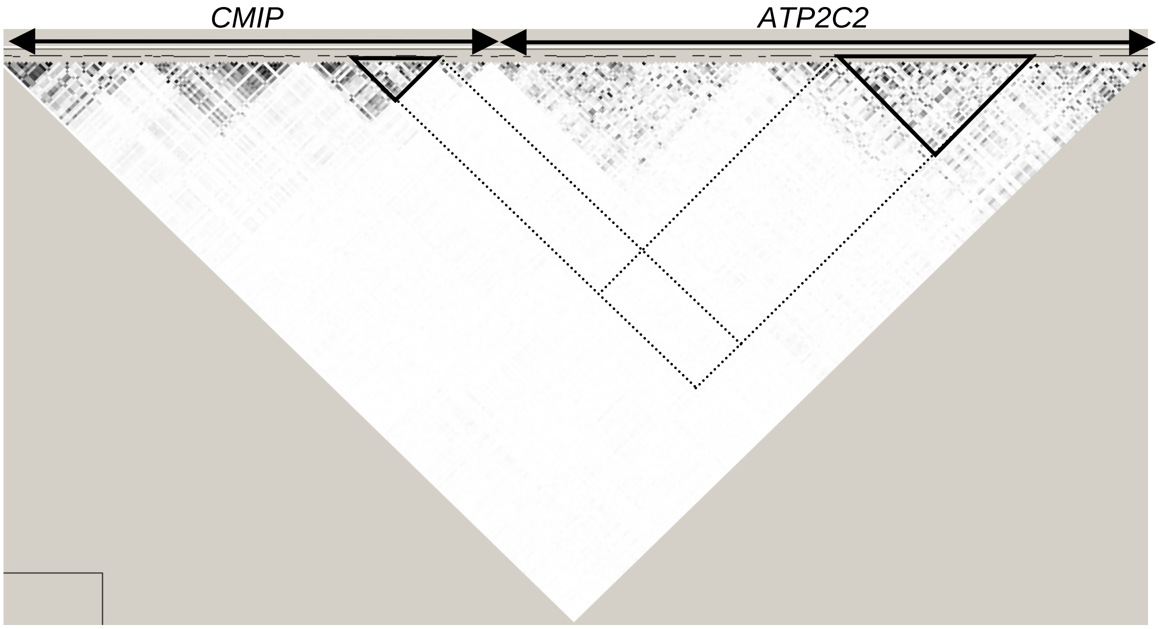

FIGURE S3 – Effects of *CMIP* and *ATP2C2* covariates upon linkage and association

The contribution of specific SNP variants to the level of linkage and association seen in the SLIC cohort was assessed by calculating the number of putative risk alleles carried at rs6564903 (*CMIP*) or rs11860694 (*ATP2C2*) for each individual. These factors were then included independently as covariates within the linkage or association model as appropriate.

Linkage was assessed within MERLIN using a variance components model with nonword repetition as a quantitative measure. Association analyses were repeated within QTDT and PLINK as appropriate.

a – The effect of *CMIP* and *ATP2C2* covariates upon linkage

It can be seen that the 2 covariates affect opposite ends of the linkage peak. Whilst the addition of a *CMIP* covariate reduces the linkage at the distal end of the peak, the *ATP2C2* covariate reduces that at the proximal part of the linkage peak.

b – The effect of *CMIP* and *ATP2C2* covariates upon association

Quant – quantitative analysis. CC – case-control analysis. Each association analysis for each SNP is shown with no covariates (NoCov), a CMIP covariate (CMIPcov, rs6564903) and an ATP2C2 covariate (ATP2C2cov, rs11860694). The *CMIP* covariate decreases the evidence for association at *CMIP* SNPs but does not have such an effect upon *ATP2C2* SNPs and the *ATP2C2* covariate decreases association at *ATP2C2* associated SNPs but not at *CMIP* associated SNPs.

FIGURE S3 cont. – Effects of *CMIP* and *ATP2C2* covariates upon linkage and association

a

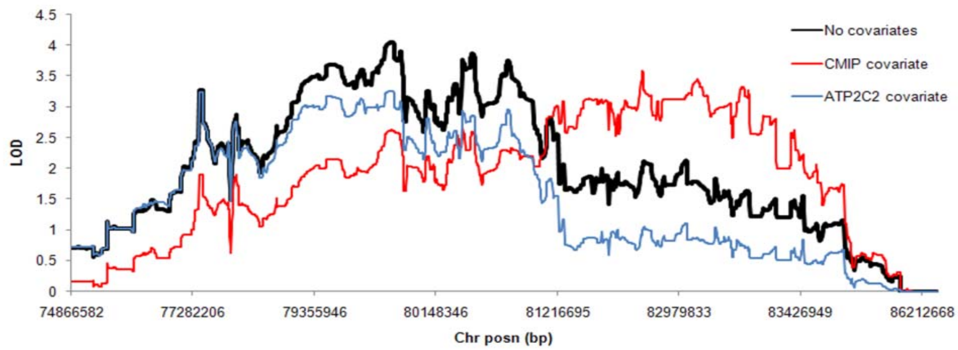

b

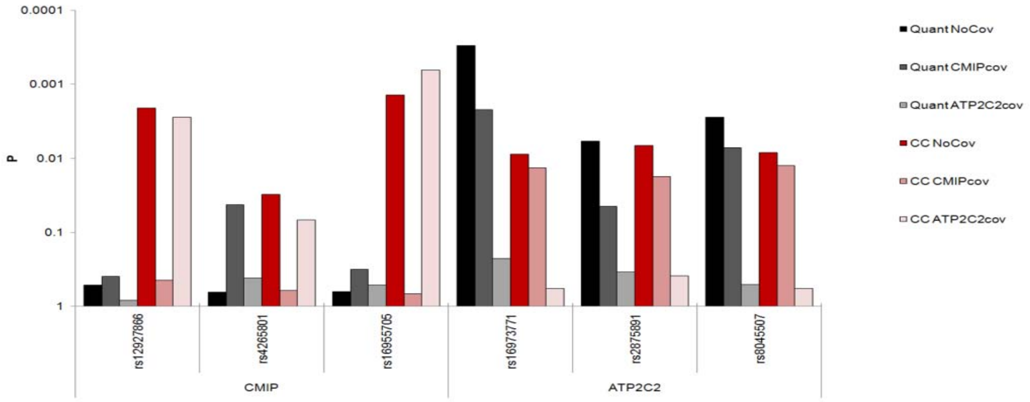

TABLE S1 – SNP coverage gene by gene

For each gene, the size of the gene, the number of SNPs typed across the gene and the average SNP spacing within the gene are given

Gene	Size (bp)	Total No SNPs	Average SNP Spacing (bp)
CNTNAP4	288,960	75	3853
NUDT7	26,744	15	1783
VAT1L	198,519	88	2256
CLEC3A	16,556	4	4139
WWOX	1,120,014	99	11313
MAF	13,877	3	4626
DYNLRB2	16,688	6	2781
CDYL2	207,500	70	2964
C16orf61	37,802	11	3437
CENPN	23,385	7	3341
ATMIN	18,494	4	4624
C16orf46	30,771	7	4396
GCSH	21,052	2	10526
PKD1L2	126,492	72	1757
BCMO1	59,452	19	3129
GAN	72,231	26	2778
CMIP	273,593	146	1874
PLCG2	185,970	92	2021
HSPC105	20,843	5	4169
HSD17B2	70,281	19	3699
MPHOSPH6	29,063	7	4152
CDH13	1,176,622	75	15688
HSBP1	12,002	4	3001
MLYCD	24,058	4	6015
OSGIN1	24,266	6	4044
NECAB2	41,143	12	3429
SLC38A8	39,374	21	1875
MBTPS1	70,149	26	2698
HSDL1	29,877	4	7469
LRRC50	39,660	11	3605
TAF1C	16,202	6	2700
ADAD2	13,029	7	1861
KCNG4	24,534	13	1887
WFDC1	42,050	22	1911
ATP2C2	102,661	150	684
KIAA1609	35,321	21	1682
COTL1	59,466	44	1352
KLHL36	20,786	3	6929
USP10	86,973	36	2416
CRISPLD2	96,530	49	1970
ZDHHC7	44,070	10	4407

TABLE S1 cont – SNP coverage gene by gene

Gene	Size (bp)	Total No SNPs	Average SNP Spacing (bp)
KIAA0513	73,419	28	2622
FAM92B	21,150	5	4230
KIAA0182	71,782	14	5127
GINS2	18,308	5	3662
C16orf74	50,566	7	7224
COX4NB	27,916	8	3490
COX4I1	14,412	1	14412
IRF8	30,438	19	1602
LOC732275	20,830	10	2083
FOXF1	10,938	2	5469
MTHFSD	32,060	13	2466
FOXC2	8,679	1	8679
FOXL1	10,190	1	10190
FBXO31	61,422	14	4387
MAP1LC3B	19,579	1	19579
ZCCHC14	92,609	14	6615
JPH3	102,263	32	3196
LOC100129637	14,542	8	1818

TABLE S2 - Association of *CMIP* and *ATP2C2* with additional language measures

Mean, standard deviations and correlation to nonword repetition (NWRcorr) are given for each trait within both the SLIC and replication samples. The correlation between sib pairs (Sibcorr) is also given for the SLIC samples. A single marker was analysed for *ATP2C2* (rs16973771) and *CMIP* (rs4265801) for each cohort using a family-based quantitative association analysis for the SLIC cohort and an allelic test of quantitative association for the ALSPAC replication cohort.

NT= not tested

CCC – Children's Communication Checklist. The pragmatic composite is provided by the sum of the inappropriate initiation, coherence, stereotyped conversation, use of context and rapport scales.

WISC - Wechsler Intelligence Scale for Children. The verbal and non-verbal IQ scores are composites of the verbal and non-verbal subtests respectively. The full IQ score is derived from all subtests.

CELF - Clinical Evaluation of Language Fundamentals. The receptive language score and expressive language score are the sum of the receptive and expressive subtests respectively. These composites can then be added to form a Total Language Score.

NWR – nonword repetition. Note that SLIC individuals completed a 28-item nonword repetition test (population mean, 100, SD, 15) whilst ALSPAC individuals completed a shortened 12-item version of this NWR test (hence the variation in means).

WOLD - Wechsler Objectives of Language Dimensions.

Note that the reading tests used by each cohort differed (hence the variation in means). The SLIC cohort completed the Wechsler Objective Reading Dimensions (WORD), while ALSPAC completed the Nunes and Bryant reading test.

TABLE S2 cont. - Association of *CMIP* and *ATP2C2* with additional language measures

			SLIC							ALSPAC					
Battery	Scale	Test	N	Mean	SD	Sib Corr	NWR corr	ATP2C2 assoc	CMIP assoc	N	Mean	SD	NWR corr	ATP2C2 assoc	CMIP assoc
CCC		Pragmatic (Linguistic) composite	103	141.08	14.97	0.11	0.34	0.7093	-0.2137	658	141.16	5.89	0.05	0.5278	-0.4233
CCC	Linguistic	Coherence	101	30.05	5.14	0.03	0.35	0.5792	-0.3115	658	33.57	2.29	0.08	0.7756	0.8538
CCC	Linguistic	Use of conversational context	100	27.70	3.70	-0.06	0.28	0.9793	-0.1241	658	27.72	2.11	0.04	0.3394	-0.2954
CCC	Linguistic	Conversational rapport	103	30.50	3.40	0.04	0.31	0.6979	0.2836	658	31.19	2.39	-0.01	-0.2916	-0.1287
CCC	Linguistic	Inappropriate initiation	102	26.61	2.95	0.44	0.12	0.3148	0.2273	658	24.39	2.72	0.04	0.5905	0.6755
CCC	Linguistic	Speech	101	30.54	4.94	-0.13	0.61	0.7417	0.5000	656	35.07	2.11	0.14	-0.1292	-0.9686
CCC	Linguistic	Sterotyped Conversation	103	26.39	3.35	0.22	0.17	0.6690	0.5353	658	24.28	2.46	-0.02	0.3919	-0.8555
CCC	Linguistic	Syntax	75	29.92	2.40	0.08	0.42	0.8974	-0.8995	657	31.68	0.65	0.07	-0.8347	-0.5602
CCC	Non-linguistic	Interests	101	31.43	2.11	0.10	-0.07	0.5203	0.7843	NT					
CCC	Non-linguistic	Social relationships	102	30.98	3.23	-0.07	0.20	0.3327	-0.4758	NT					
WISC		Full IQ	368	95.39	16.77	0.21	0.36	-0.1321	-0.5501	492	103.26	13.94	0.26	-0.1479	-0.6764
WISC		Verbal IQ	368	94.45	17.02	0.20	0.43	-0.0805	-0.9328	494	104.55	15.62	0.31	-0.4648	-0.773
WISC	Verbal	Arithmetic	353	8.73	3.56	0.16	0.38	-0.1976	-0.9466	495	10.05	3.86	0.25	-0.8531	-0.2074
WISC	Verbal	Comprehension	353	8.44	3.37	0.08	0.33	**-0.0328**	0.8585	491	10.75	3.71	0.08	0.8965	-0.8899
WISC	Verbal	Digit span	352	7.80	3.15	0.17	0.49	-0.0836	0.7891	484	9.90	2.99	0.36	-0.3878	-0.4395
WISC	Verbal	Information	327	9.15	3.68	0.21	0.39	-0.1123	0.8838	495	10.66	3.13	0.26	-0.9564	0.4793
WISC	Verbal	Similarities	353	9.78	3.55	0.30	0.37	-0.1481	0.8208	495	11.86	3.97	0.22	-0.3697	0.6119
WISC	Verbal	Vocabulary	353	8.79	3.18	0.25	0.35	**-0.0399**	-0.8050	492	10.53	4.17	0.27	-0.3041	-0.5808
WISC		Performance (nonverbal) IQ	385	97.76	17.54	0.17	0.20	-0.5433	-0.3097	492	100.89	14.47	0.08	-0.08312	-0.5608
WISC	Performance	Block design	370	9.67	3.38	0.29	0.14	-0.7783	-0.2441	493	11.01	3.63	0.16	0.2880	-0.9002
WISC	Performance	Coding	344	8.89	3.38	0.14	0.26	-0.0744	0.3330	494	10.39	2.79	0.04	-0.3219	0.7197
WISC	Performance	Object assembly	343	9.38	3.04	0.18	0.15	-0.9593	-0.3631	463	10.28	3.65	0.04	-0.08437	-0.2592
WISC	Performance	Picture arrangement	344	10.05	3.74	0.09	0.12	0.2627	-0.5957	491	9.81	4.62	-0.09	-0.08769	0.5036
WISC	Performance	Picture completion	370	9.90	2.86	0.14	0.11	0.8911	-0.6634	493	9.23	3.44	0.11	-0.3290	-0.1881
WISC	Memory	Mazes	334	9.20	3.64	0.16	0.13	0.0713	0.7868	NT					
WISC	Memory	Symbols	340	9.54	3.41	0.19	0.26	-0.1505	0.8106	NT					

TABLE S2 cont. - Association of *CMIP* and *ATP2C2* with additional language measures

			SLIC							ALSPAC					
**Battery**	**Scale**	**Test**	**N**	**Mean**	**SD**	**Sib Corr**	**NWR corr**	**ATP2C2 assoc**	**CMIP assoc**	**N**	**Mean**	**SD**	**NWR corr**	**ATP2C 2 assoc**	**CMIP assoc**
CELF		Total language score	441	81.39	16.76	0.20	0.53	**-0.0352**	0.0963	NT					
CELF		Expressive language composite	442	77.64	15.50	0.15	0.55	-0.0918	-0.254	NT					
CELF	Expressive	Formulating sentences	428	5.62	2.52	0.14	0.40	**-0.0395**	-0.6832	NT					
CELF	Expressive	Recalling sentences	428	6.77	3.09	0.24	0.56	-0.0592	-0.1627	NT					
CELF	Expressive	Sentence assembly	359	8.09	2.57	0.20	0.31	-0.7951	-0.0763	NT					
CELF		Receptive language composite	442	87.21	18.00	0.21	0.47	**-0.0449**	0.0805	NT					
CELF	Receptive	Oral directions	429	8.10	3.13	0.13	0.45	**-0.0064**	0.0535	NT					
CELF	Receptive	Semantic relations	360	8.61	3.16	0.28	0.40	-0.408	0.3878	NT					
CELF	Receptive	Word classes	364	8.19	3.18	0.12	0.41	**0.0446**	0.2339	NT					
NWR		Total nonword repetition	503	91.48	20.11	0.38		**-0.0003**	0.3446	491	6.87	2.41		**-0.0079**	**-0.0182**
NWR	Syllables	Nonword repetition, 3 syllables	NT							491	2.86	1.00	0.68	-0.1894	-0.09041
NWR	Syllables	Nonword repetition, 4 syllables	NT							491	2.12	1.09	0.76	-0.3312	-0.2695
NWR	Syllables	Nonword repetition, 5 syllables	NT							491	1.88	1.16	0.78	**-0.0006**	**-0.0155**
		Counting span	NT							475	3.34	0.85	0.22	**-0.0143**	-0.3282
WOLD		Listening comprehension	NT							494	6.84	2.36	0.16	-0.2084	-0.3005
WORD/ N&B		Single word reading	345	92.20	15.68	0.30	0.56	-0.2907	-0.2969	548	26.81	9.14	0.45	-0.4366	0.6713
WORD		Reading comprehension	198	90.48	17.46	0.46	0.48	-0.4176	0.9815	NT					
WORD/ N&B		Single word spelling	341	90.96	16.03	0.28	0.52	-0.1116	0.6939	543	6.92	4.29	0.33	0.8855	-0.7507
